# The PGE2–EP receptor axis in urinary tract smooth muscle: one signaling system, three organ-specific strategies

**DOI:** 10.3389/fphys.2026.1977562

**Published:** 2026-10-28

**Authors:** Hao Su, Wenyan Zhou, Fei Ao, Tianrong Ye, Ganhua You, Dongbo Yuan, Jianguo Zhu, Houyong Zhou

**Affiliations:** 1 Department of Urology, the Second People's Hospital of Guizhou Province, Guiyang, Guizhou, China; 2 Department of Laboratory Medicine, Guizhou Branch of Shanghai Children's Medical Center, Shanghai Jiao Tong University School of Medicine, Guiyang, Guizhou, China; 3 Department of Scientific Research, the Second People's Hospital of Guizhou Province, Guiyang, Guizhou, China; 4 Department of Urology, Guizhou Provincial People's Hospital, Guiyang, Guizhou, China

**Keywords:** detrusor, EP receptor, overactive bladder, prostaglandin E2, ureteral peristalsis, urinary tract smooth muscle

## Abstract

Prostaglandin E2 (PGE2) is the dominant lipid mediator generated within the urinary tract wall, yet the direction of its action reverses from organ to organ: it excites the bladder, suppresses the ureter, and relaxes the prostate and urethra. This review asks why one signaling system produces three opposite outputs and what that organization offers for therapy. Synthesizing 115 publications, from classical organ-bath studies to current clinical trials, we first outline the molecular toolkit—an inducible biosynthetic arm and four EP receptor subtypes with asymmetric G protein coupling—that makes divergent deployment chemically possible. We then assemble the organ-specific evidence. In the bladder, EP1/EP3-driven contraction and afferent sensitization are distributed across a chimera of smooth muscle, urothelium, interstitial cells, and nerves, and are amplified by obstruction, inflammation, denervation, and aging. In the ureter, EP2-mediated inhibition of peristalsis—with the EP4 component still an inference from coupling logic—is the property that NSAIDs, the first-line analgesics for renal colic, act upon, although whether their benefit arises from restoring motility, reducing edema, or dampening nociceptors remains unresolved. In the outflow tract, the weakest-evidenced arm, EP-dependent relaxation is supported by rodent pharmacology and by a single clinical association between pathway activation and BPH that fails medical therapy. Within this PGE2-centered frame, receptor-subtype repurposing across organs is consistent with rules that pathology reweights but does not erase—weights inferred from function rather than measured receptor abundance—and much of the literature’s apparent contradiction dissolves under five methodological variables. We propose the axis as one system deployed in three organ-specific strategies—a testable working hypothesis, with the outflow tract currently a plausible third member, under which choosing a receptor subtype would approximate choosing an organ. The hypothesis maps onto therapy as one established success, two credible prospects—EP2/EP3 agonism for underactive bladder and EP2 agonism for stone expulsion—and one open frontier, and we define the maps, conditional knockouts, verified ligands, and computational tools needed to convert it into organ-directed pharmacology.

## 1 Introduction

Smooth muscle tone along the urinary tract determines whether the bladder stores urine at low pressure, the ureter propels it without pain, and the bladder outlet opens on demand. When these processes fail, highly prevalent disorders follow. Overactive bladder (OAB) affects roughly one in nine adults worldwide (Zhang et al., 2025; Irwin et al., 2006). Symptomatic kidney stones occur in about one in eleven individuals (Scales et al., 2012). Symptoms of benign prostatic hyperplasia (BPH) trouble a large proportion of men over 60 (Egan, 2016), and among adults with OAB, women carry the larger share (Zhang et al., 2025). Pharmacological control of urinary smooth muscle remains incomplete: antimuscarinics and β3-adrenoceptor agonists provide only moderate benefit in OAB, while the analgesic mainstay for renal colic, non-steroidal anti-inflammatory drugs (NSAIDs), is prescribed largely on empirical grounds.

Prostaglandin E2 (PGE2) is the principal lipid mediator produced within the urinary tract wall. It is synthesized from membrane phospholipids through cyclooxygenase-1 and -2 (COX-1/COX-2) together with microsomal PGE synthase-1. It then signals through four G protein-coupled receptor subtypes, EP1 to EP4, defined pharmacologically by the International Union of Pharmacology (Coleman et al., 1994; Breyer et al., 2001). Their coupling is asymmetric: EP1 drives Gq-mediated Ca²^+^ mobilization, EP2 and EP4 drive Gs-mediated cAMP accumulation, and EP3 acts primarily through Gi. In human urinary tract tissue, infection and obstruction induce COX-2 and augment PGE2 production, tying inflammation to altered smooth muscle behavior (Wheeler et al., 2002).

Research on this mediator in the urinary tract spans six decades. The field opened in 1966 with the observation that prostaglandins inhibit ureteral peristalsis (Boyarsky et al., 1966), followed by experiments on isolated human bladder and urethra in the late 1970s showing that PGE2 raises intravesical pressure while relaxing urethral tissue (Andersson et al., 1977, Andersson et al., 1978a). A 1995 study established the conscious-rat model of PGE2-induced bladder overactivity that remains in wide use (Ishizuka et al., 1995). Genetic experiments then assigned function to individual subtypes: EP1-deficient mice show altered detrusor responses to PGE2 under bladder outlet obstruction (BOO) (Schröder et al., 2004), and EP3-deficient mice display enlarged bladder capacity with loss of PGE2-evoked hyperactivity (McCafferty et al., 2008). Recent work has extended the mechanism to interstitial cells (EP3 activation enhances bladder excitability through HCN channels on interstitial cells of Cajal (Wu et al., 2017)) and to the clinic, where PGE2–EP2 signaling relaxes the ureter and promotes stone expulsion (Su et al., 2024).

Despite this accumulated knowledge, the literature remains fragmented along organ lines. The most cited review of prostanoids in bladder physiology is now more than a decade old and, by design, covers all five prostanoids within a single organ (Rahnama’i et al., 2012a). A more recent appraisal of PGE2 receptors concentrates on the detrusor from a drug-development perspective (Hou et al., 2021), and ureter-directed reviews confine themselves to the pharmacology of that one segment (Canda et al., 2007). No available synthesis asks why a single mediator produces excitation in the bladder, inhibition in the ureter, and relaxation in the prostate and urethra.

This review addresses that question directly, with the strength of the answer stated at the outset rather than deferred. Drawing on 115 landmark and closely related studies, we examine the hypothesis that the PGE2–EP axis operates as one signaling system deployed in three organ-specific strategies. EP1/EP3-driven excitation and afferent sensitization govern the bladder. EP2-mediated inhibition of ureteral peristalsis—with EP4 participation still an inference from coupling logic—is the property NSAIDs exploit in renal colic. EP-dependent relaxation plausibly governs the prostate and urethra. Cross-species comparisons that include human tissue data (Root et al., 2015) indicate that such receptor-subtype repurposing is reproducible enough to be treated as an organizing framework rather than a collection of organ-specific curiosities. The three rows of that framework do not rest on equal evidence: the bladder assignment draws on knockout genetics and human pharmacology, the ureteral assignment on a single mechanistic animal study plus clinical analogy, and the outflow assignment on rodent pharmacology plus one clinical association, so the third strategy in particular is a well-supported hypothesis rather than an established mechanism. We first outline the molecular toolkit of PGE2 synthesis and EP signaling (Section 2), then examine each organ in turn (Sections 3–5), develop a comparative framework and analyze the methodological sources of discordant findings (Section 6), and close with therapeutic implications and tractable research gaps (Sections 7, 8).

The synthesis draws on a documented search strategy (**Supplementary Figure S1**). We searched PubMed on 27 September 2026 (coverage 1966–2026, English-language records) with a fixed string combining pathway terms (prostaglandin E2, PGE2, EP receptor, E-prostanoid, EP1, EP2, EP3, EP4, cyclooxygenase-2, COX-2, microsomal prostaglandin E synthase, mPGES-1) with organ terms (bladder, detrusor, ureter, renal pelvis, pyeloureteral, prostate, prostatic, urethra, urinary tract, urothelium) in title and abstract fields; the query is reproduced verbatim in the **Supplementary Material**. The search returned 2,053 records, 1,977 after language and date filters. Records were screened at the title and abstract level for primary experimental or clinical studies of PGE2 synthesis, EP receptor signaling, or prostaglandin-directed intervention in urinary tract smooth muscle; reviews were retained only where they framed the structure of the field, and case reports and studies without urinary tract smooth muscle relevance were excluded. The final evidence base of 115 references assembles 76 records retrieved by the database string with 39 added by reference mining of landmark reports; screening was iterative rather than prospectively logged, in keeping with a narrative review. The reference list has been reconciled against this count entry by entry.

## 2 PGE2 biosynthesis and EP receptor signaling: the molecular toolkit

The PGE2 signaling system is built for versatility. Its biosynthetic arm separates resting from inflamed states, so that mediator output can rise within hours of tissue stress; its receptor arm comprises four G protein-coupled subtypes with non-overlapping coupling preferences, so that a single lipid mediator can raise calcium in one cell while raising cAMP in its neighbor. This dual architecture, an inducible supply coupled to asymmetric reception, is what makes organ-specific deployment of the same signal chemically plausible. The present section outlines the three components of the toolkit in turn: the biosynthetic enzymes, the receptor–effector map, and the subtype-selective ligands through which each element has been tested in urinary tract tissue.

PGE2 synthesis in the urinary tract wall depends on an inducible two-enzyme arm whose output rises sharply during infection, obstruction, and mechanical stress. Arachidonic acid released from membrane phospholipids is converted by cyclooxygenase to PGH2, the committed precursor, and terminal synthesis of PGE2 is executed largely by microsomal PGE synthase-1, an inducible enzyme functionally coupled to COX-2 (Sampey et al., 2005; **Figure 1**). In the bladder, COX-2 is expressed during fetal development and reappears when the outlet is obstructed, where the induction proceeds on a timescale of hours rather than days (Park et al., 1997); in human urinary tract tissue obtained during infection, COX-2 induction with increased PGE2 production has been documented directly (Wheeler et al., 2002). The practical consequences of this inducible organization are twofold. First, basal PGE2 output in healthy tissue is low, which helps explain why NSAIDs alter urinary tract behavior mainly under inflammatory or obstructive conditions rather than at rest. Second, suppressing the pathway at one node invites compensation at another: mice lacking mPGES-1 redirect accumulated precursor toward prostacyclin, altering cardiovascular outcomes while preserving much PGE2-dependent physiology (Wang et al., 2006). Any strategy built on synthesis blockade rather than receptor-level targeting must reckon with such metabolite re-routing.

**Figure 1:**
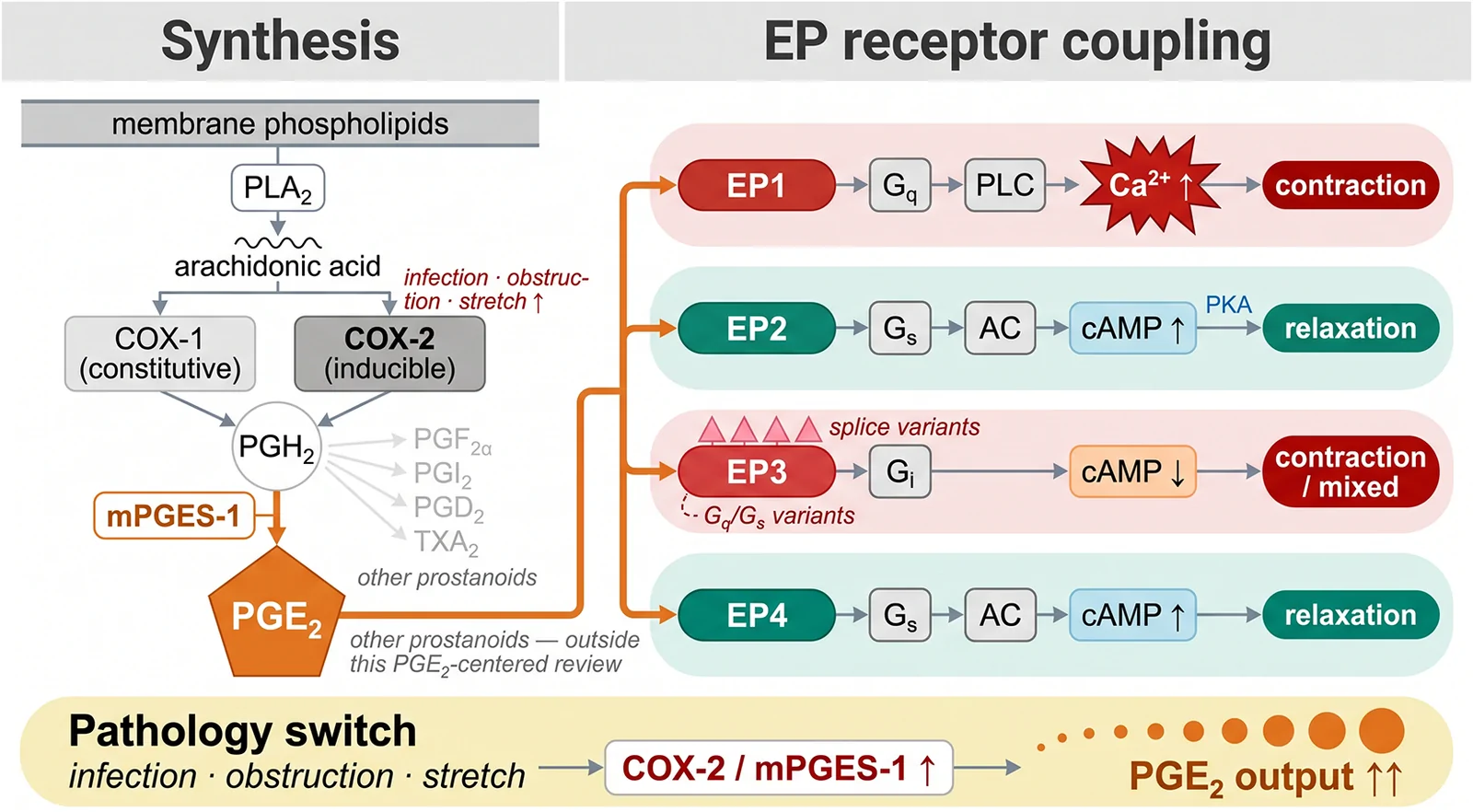
PGE2 biosynthesis and asymmetric EP receptor signaling in the urinary tract wall. Left: PGE2 synthesis runs from arachidonic acid through cyclooxygenase to PGH2, with terminal synthesis by microsomal PGE synthase-1 (mPGES-1), an inducible enzyme coupled to COX-2. Right: the four EP subtypes couple to non-overlapping G proteins—EP1 via Gq–PLC to Ca2+ increase, EP2 and EP4 via Gs to cAMP accumulation, EP3 mainly via Gi to cAMP reduction, with splice variants that can engage Gq or Gs—so one mediator can raise calcium in one cell and cAMP in its neighbor. Bottom: infection, obstruction, and mechanical stress upregulate COX-2 and mPGES-1, amplifying mediator output. EP, E-prostanoid receptor; PLA2, phospholipase A2; AC, adenylyl cyclase; PLC, phospholipase C; PKA, protein kinase A.

Signal divergence originates at the receptor level. The four EP subtypes were defined pharmacologically by the International Union of Pharmacology (Coleman et al., 1994). Each couples to a distinct G protein family. EP1 engages Gq, turning over phosphoinositide and mobilizing calcium. EP2 and EP4 raise intracellular cAMP through Gs. EP3 is the most plastic of the four: its gene yields multiple splice variants with divergent carboxy-terminal tails, most of them Gi-coupled and cAMP-lowering, while some variants acquire Gs or Gq competence (Breyer et al., 2001). The variants are not pharmacological trivia: distinct EP3 isoforms can route the same ligand into Gi, Gs, or Gq signaling within one species, which gives an organ the option of re-tuning its response to PGE2 without altering receptor expression at all (Breyer et al., 2001). Two features of this map deserve emphasis for what follows. One is directional asymmetry: calcium mobilization in smooth muscle is conventionally excitatory, whereas cAMP accumulation is relaxant, so the EP1/EP3 versus EP2/EP4 split provides a ready molecular basis for contraction and relaxation arising from one ligand. The other is cell-type dependence. Pharmacological characterization of isolated human detrusor has demonstrated coexisting prostanoid receptor populations with distinct agonist potencies within the same tissue (Palea et al., 1998), which means that the net response to PGE2 in an organ reflects the weighted sum of its cellular reception rather than a fixed receptor property. Pathological experience reinforces the point: in a rat model of non-bacterial prostatic inflammation, EP receptor expression in the bladder mucosa shifts and EP4 blockade attenuates the resulting bladder overactivity, so receptor function cannot be read as a static property of the healthy wall (Mizoguchi et al., 2019).

Assigning function to individual subtypes in urinary tract tissue has relied on a gradually assembled toolbox of subtype-selective ligands. On the antagonist side, compounds with meaningful EP4 selectivity have been characterized urodynamically in rodents, where they reduce detrusor overactivity and shed light on EP4’s contribution to bladder excitability (Chuang et al., 2010, Chuang et al., 2012). On the agonist side, ONO-8055, a dual EP2/EP3 agonist, has demonstrated pro-voiding activity across multiple models of the underactive bladder (UAB) (Sekido et al., 2016; Matsuya et al., 2018; Sekido et al., 2020a); Section 7 takes up its therapeutic record in full. The available subtype-selective ligands, agonists and antagonists alike, are assembled in **Table 1**. The availability of such tools explains why organ-level questions can now be asked with subtype resolution. The same tools also expose a limitation: most were characterized in rodents, and their selectivity profiles do not always transfer to human tissue, where receptor pharmacology has been mapped in detail for the detrusor but far less thoroughly for ureter and prostate (Palea et al., 1998).

**Table 1:** EP receptor subtypes: coupling, second messengers, and representative subtype-selective tool ligands.

Receptor	G protein coupling	Second messenger	Net effect on smooth muscle	Selective/used antagonists (context)	Selective/used agonists (context)
EP1	Gq → PLC	Ca²^+^ ↑	Contraction	PF-2907617-02 (rat urodynamics)	—
EP2	Gs → AC	cAMP ↑	Relaxation	(within AH6809 dual EP1/EP2, porcine strips)	ONO-8055 (dual EP2/EP3)
EP3	Gi (variants: Gs/Gq)	cAMP ↓ (variant-dependent)	Contraction/mixed	DG-041 (rat afferent studies); L-798106 (porcine strips)	— (see EP2 row: ONO-8055 is dual EP2/EP3)
EP4	Gs → AC	cAMP ↑	Relaxation	AH23848 (rat OAB); MF191 (rat OAB); ASP7657 (systemic safety)	—

Ligand selectivity established primarily in rodents; human-tissue verification remains partial (see text). Antagonist entries list the ligand with the experimental context in which it was used in the cited studies; a dash indicates that no subtype-selective agonist for that receptor appears in the cited literature. The agonist column is restricted to ligands deployed in the urinary tract studies cited in this review—ONO-8055 (a dual EP2/EP3 agonist) is therefore listed once, under EP2—whereas subtype-selective EP1 and EP4 agonists exist as pharmacological tools (e.g., ONO-DI-004, ONO-AE1-329) but have not been characterized in urinary tract tissue in the literature synthesized here.

Taken together, the toolkit defines both the reach and the boundaries of current knowledge. At the verification level, whether the subtype assignments established in rodents hold across human detrusor, ureter, and prostate is only partially tested; human pharmacology is most complete for the detrusor, yet quantitative receptor mapping in ureteric and prostatic tissue remains sparse. At the atlas level, no organ-resolved and cell-resolved expression map of EP1–EP4 is available for the urinary tract, so quantitative comparison among organs still rests on inference from function rather than on measured transcript or protein abundance. At the translational level, the mPGES-1 lesson (metabolite re-routing after synthesis blockade) argues for receptor-level targeting, and the ligand toolbox is the bridge by which such targeting can be attempted. The molecular machinery thus permits, in principle, three different organ strategies; whether the urinary tract actually employs them is the subject of the next three sections.

## 3 The bladder: EP1/EP3-driven excitation and sensitization

The bladder deploys the PGE2–EP axis for excitation on two fronts, motor and sensory, and it does so through a chimera of receptor-bearing cell types rather than through the detrusor alone. Smooth muscle from most species contracts to PGE2 through EP1- and EP3-like receptors; the urothelium that lines the cavity both generates the mediator and expresses its receptors; interstitial cells of Cajal (ICC) provide an amplifier for spontaneous activity; and primary afferents are sensitized within minutes of exposure. Each of these four cellular stations contributes a distinct component to bladder overactivity, which is why the field has moved from organ-level pharmacology toward cell-resolved mechanisms over the past two decades. This section follows that trajectory: from receptor distribution, through the disputed direct effects on detrusor strips, to the interstitial-cell pacemaker route, the afferent arm, and finally the phenotypic reprogramming documented in obstruction, inflammation, denervation, and aging.

EP receptors are distributed across every functional layer of the bladder wall (**Figure 2**). In the smooth muscle compartment, comparative *in vitro* work across rat, macaque, and human tissue has established that contractile prostanoid responses are mediated principally by EP1- and EP3-like receptors, with contraction ranking among the most robust prostanoid effects in human detrusor (Root et al., 2015). The mucosa contributes its own reception: binding sites for EP1 and EP2 have been localized to guinea pig urothelium and lamina propria (Rahnama’i et al., 2010), and EP2 receptor distribution has been mapped in the guinea pig detrusor (Rahnama’i et al., 2012b). Intramural ganglia of the bladder wall express EP receptors as well, positioning PGE2 to modulate local neural integration before signals ever reach the spinal cord (Rahnama’i et al., 2015). A fourth station is formed by interstitial cells of Cajal, identified in the human bladder and proposed early on as vesical pacemakers (Shafik et al., 2004); their spontaneous activity is well documented by modern imaging, even though their necessity for pacing remains debated (Gupta et al., 2024). The organ, in short, is a mosaic of reception sites, and its response to PGE2 should be read as the sum of that mosaic.

**Figure 2:**
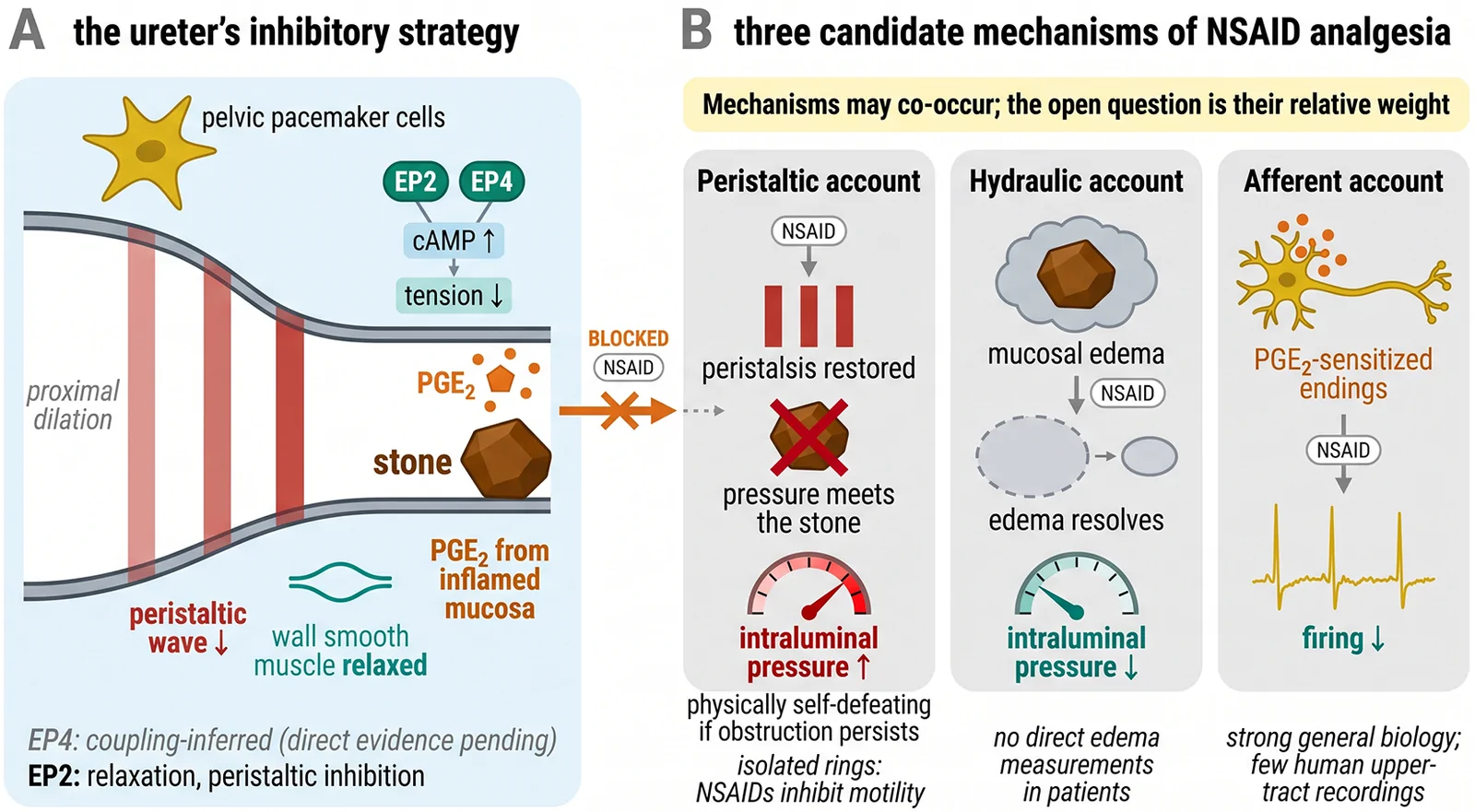
The bladder wall as a receptor chimera: motor and sensory arms of PGE2 signaling. Schematic cross-section (lumen top). (1) The urothelium senses stretch and releases ATP and PGE2; EP1/EP2 sites mark urothelium and lamina propria. (2) EP3 reception on interstitial cells of Cajal engages HCN channels, generating spontaneous depolarization; cyclophosphamide upregulates HCN1 with parallel overactivity. (3) Detrusor contraction proceeds mainly through EP1-/EP3-like receptors and Ca2+ mobilization, with a low-dose relaxant limb (EP2/EP4). (4) Afferent endings are sensitized by EP3 reception and urothelial ATP/PGE2. Right: motor (non-voiding contractions) and sensory (urgency) outputs converge on the overactive bladder phenotype; capsaicin desensitization spares the intravesical PGE2 response, implicating a non-neural, likely mucosal, step. Pathological triggers—BOO, inflammation, SCI, and aging—induce COX-2 and re-express EP4, amplifying the chimera. ICC, interstitial cells of Cajal; HCN, hyperpolarization-activated cyclic nucleotide-gated; BOO, bladder outlet obstruction; SCI, spinal cord injury.

The direct action of PGE2 on detrusor strips is biphasic and species-dependent, an unresolved pattern that continues to generate contradictory reports. Early comparative work recorded relaxation and contraction within the same preparations: PGE2, PGF2α, and prostacyclin each left distinct signatures on bladder versus urethral tissue (Gotoh et al., 1986). Field-stimulation experiments in rabbit detrusor showed prostaglandins participating in neurogenically evoked contractions (Downie and Larsson, 1981). The mediator therefore sits at the neuroeffector junction, not only at the muscle surface. Comparing PGE2 with sulprostone, an agonist acting mainly at EP1 and EP3, revealed divergent response patterns decades before knockout mice existed: the two ligands did not overlap, implying that contraction and relaxation ride on different receptors (Schüssler, 1990). In rabbit detrusor, PGE2 mediates spontaneous rhythmic contraction, tying the mediator to intrinsic activity, not evoked tone (Klausner et al., 2011), a conclusion reinforced by evidence that bladder interstitial cells express cyclooxygenase and that COX products drive the spontaneous rhythmic contraction of isolated rabbit bladder strips (Collins et al., 2009). Layer-resolved experiments show that PGE2 and PGF2α act differently on urothelium, lamina propria, and detrusor, so that stripping the mucosa changes the apparent contractile response (Stromberga et al., 2020). Aging adds another dimension, shifting detrusor reactivity to prostaglandins and other agonists (Phelps et al., 2023). No single concentration–response model reconciles these variables across species and preparations; the pattern awaits the systematic methodological treatment given in Section 6.

Spontaneous detrusor activity links PGE2 to interstitial cells: EP3 activation raises bladder excitability through HCN channels on interstitial cells of Cajal (Wu et al., 2017). The finding matters because it supplies a mechanistic route from inflammatory mediator to organ-level hyperexcitability that bypasses the nerve terminal. Supporting evidence comes from the inflammation side of the ledger: cyclophosphamide upregulates HCN1 in bladder interstitial cells and produces overactivity that tracks the channel change (Liu et al., 2017). Two findings bracket the mechanism at both ends: the PGE2 dependence of spontaneous rhythmic contraction in rabbit detrusor (Klausner et al., 2011), and the identification of pacing-competent interstitial cells in human bladder (Shafik et al., 2004). The debate over whether interstitial cells are necessary pacemakers or modulators still lingers (Gupta et al., 2024), and that keeps the interpretation honest. On current evidence, the EP3–HCN–interstitial cell axis is the best-characterized non-neural route by which PGE2 excites the bladder.

PGE2 sensitizes bladder afferents, and this sensory arm can be dissociated from the motor arm. Direct recordings show that the mediator augments afferent firing during bladder distension (Kuga et al., 2016). Intravesical PGE2 in conscious rats produces bladder hyperactivity, the founding observation of the standard animal model of overactive bladder (Ishizuka et al., 1995), and in the PGE2 cat model, stimulation of the pelvic nerve increases bladder capacity, indicating that the sensory route is open to modulation (Langdale et al., 2020). The dissociation itself was demonstrated in the acute rat model, where PGE2 evoked the symptom profile of overactivity without detrusor overactivity during filling (Hokanson et al., 2017). Which cellular element carries the sensory signal remains an open question with a partial answer: after capsaicin desensitization, the effects of intravesical PGE2 on bladder function are preserved, implicating a non-neural, likely mucosal, transduction step (Hokanson et al., 2021). The urothelium is the leading candidate for that step. Bladder distension increases urothelial ATP release (Ferguson et al., 1997), PGE2 augments this stretch-evoked release via EP1/EP3 receptors (Tanaka et al., 2011), and cytotoxic injury exaggerates the same release pathway (Kang et al., 2015). Inflammatory stimuli reshape the output more broadly, altering urothelial ATP, acetylcholine, and prostaglandin release at once (Kang et al., 2013). Antimuscarinic drugs add a therapeutic angle: they suppress urothelial ATP and PGE2 release, which is one way a drug aimed elsewhere acts partly here (Yokoyama et al., 2011). Mast cells add an upstream trigger to the same cascade: degranulation by compound 48/80 produces urothelium-dependent increases in murine bladder activity (Jones et al., 2023), which positions the mucosa as an integrator of immune and mechanical input whose prostaglandin output feeds both the afferent and the motor arms described above. The instillation model that anchors much of this work deserves its own caution. Acute intravesical PGE2 at the concentrations commonly used, 100 µM in the rat dissociation study (Hokanson et al., 2017), exceeds anything the urothelium generates physiologically. A single acute exposure also fails to reproduce the sustained, low-grade mediator output of chronic obstruction or cystitis. The model is therefore best read as a maximal pharmacological challenge that reveals the axis’s capacity, not as a replica of disease dosimetry.

Obstruction, inflammation, denervation, and aging each reprogram the receptor phenotype, converting the physiological chimera into a pathological amplifier. Under outlet obstruction, bladder COX-2 is re-induced (Park et al., 1997) and EP4 expression rises (Beppu et al., 2011); EP1-deficient mice show altered detrusor responses to PGE2 and altered development of obstruction-induced hyperactivity, placing EP1 inside the pathological cascade rather than beside it (Schröder et al., 2004). Inflammatory insults act in the same direction. Cyclophosphamide cystitis upregulates COX-2 and prostanoid production along micturition pathways (Hu et al., 2003). Human infected urinary tract tissue shows induced COX-2 with increased PGE2 output (Wheeler et al., 2002). Urothelial cells from interstitial cystitis patients release PGE2 aberrantly (Rastogi et al., 2008), and intravesical botulinum toxin suppresses COX-2 and EP4 expression together with non-voiding contractions (Chuang et al., 2009). Denervation and central injury add the neural dimension: spinal cord injury raises bladder PGE2 release (Masunaga et al., 2006), peripheral EP3 receptors excite bladder afferents (Su et al., 2008a), central EP3 receptors modulate micturition (Su et al., 2008b), and in experimental autoimmune cystitis, EP3 and EP4 receptors contribute jointly to bladder dysfunction (Xue et al., 2013). The sharpest resolution comes from genetics: EP3-deficient mice exhibit enlarged bladder capacity and lose PGE2-mediated hyperactivity (McCafferty et al., 2008), a phenotype distinct from that of EP1 deficiency (Schröder et al., 2004), demonstrating that the two excitatory subtypes divide the labor: EP3 stands closer to the afferent and pacing apparatus, EP1 to the obstruction-responsive contractile response. The reprogramming also has non-prostanoid dimensions that fix its scope: obstruction activates mechanotransductive and growth-factor signaling in bladder smooth muscle (Yamaguchi, 2004) and raises passive stiffness through the Rho kinase axis (Speich et al., 2012), so the prostaglandin axis operates as one amplifier among several, neither sufficient nor necessary for the whole hypertrophic phenotype. Placing it accurately, as the inflammatory-sensory limb of a larger remodeling process, is what keeps the therapeutic claims of Section 7 proportionate.

The bladder’s arms met the clinic early, by instillation rather than by design. When PGE2 was instilled into the bladders of women with emptying disorders, the storage organ and its outlet moved in opposite directions under one mediator, the first human demonstration of the dual-arm control documented later in animals (Andersson et al., 1978a). Instillation of PGE2 and PGF2α was then attempted for bladder emptying disorders in women (Delaere et al., 1981), and the most durable therapeutic use appeared in hematology, where PGE2 bladder instillation treated hemorrhagic cystitis after allogeneic bone marrow transplantation (Laszlo et al., 1995). These early clinical experiences were small and uncontrolled, but they established two points that animal work could not: the human bladder responds to exogenous PGE2 in the predicted direction, and the crude agonist cannot separate the storage symptoms it provokes from the emptying benefit it was seeking. That separation is precisely what subtype-selective tools are meant to achieve, which is why the therapeutic story returns in Section 7.

The bladder strategy thus resolves into a testable chain: inducible synthesis, mosaic reception, dual motor–sensory output, and pathological amplification. Translational openings already exist in outline. A β3-adrenoceptor agonist and intravesical PGE2 have been directly compared at the bladder afferent level in rats; the two stimuli interact within one pathway (Aizawa et al., 2010). In a diabetic rat model of hyposensitive underactive bladder, PGE2 and its receptors change together with bladder function. That nominates receptor competence as a candidate biomarker (Nirmal et al., 2014). Three gaps define the forward agenda. At cellular resolution, no study has directly localized EP3 protein to human bladder interstitial cells in disease, so the central non-neural mechanism rests on rodent evidence. At human validation, the subtype assignments from knockout mice await systematic testing in human detrusor across sex and age. At the intervention window, it remains unknown whether the pathological receptor reprogramming described above is reversible; the botulinum toxin experience, where COX-2 and EP4 fall together with symptoms, hints that it may be, and Section 7 takes up the pharmacological consequences.

## 4 The ureter: EP2-dominated inhibition of peristalsis

The ureter inverts the bladder strategy. Through EP2-dominated reception—an EP4 contribution is inferred from coupling logic but remains untested by direct experiment in the human organ—PGE2 dampens tone and slows peristalsis, an arrangement that serves the organ’s mission of moving urine at low pressure and that obstruction-related inflammation turns against it. Where the bladder’s EP1/EP3 deployment converts the inflammatory mediator into contractile force and afferent alarm, the ureter’s EP2-dominated converts the same mediator into motor quiet, and the clinical correlate inverts accordingly: NSAIDs, which block PGE2 synthesis, remain first-line analgesia for renal colic. This section traces the inhibitory phenotype from its classical pharmacological documentation to its recent molecular address, examines the two-faced remodeling that obstruction imposes on the pathway, and closes with the unresolved division of labor among three mechanisms of NSAID analgesia.

Ureteral peristalsis is generated by a multicellular engine whose components each carry prostaglandin reception or respond to it. Electrical recordings across mammalian species established the myogenic basis of the propagating contraction, with pacemaker activity residing in atypical smooth muscle cells of the renal pelvis (Lang et al., 1998). Interstitial cells of Cajal, the same family recruited as bladder modulators in Section 3, populate the pyeloureteral wall. They shape the frequency and propagation of the peristaltic wave (Lang and Hashitani, 2019), a pacemaker contribution reviewed in depth for the upper urinary tract (Weiss et al., 2006). In this conduit they serve rhythmic transport, not storage-triggered voiding. Sensory nerves close the loop. Capsaicin-sensitive sensory nerves are essential for normal pyeloureteral motility together with endogenous prostaglandins, so that peristalsis behaves as a neuro-myogenic hybrid rather than a pure muscle rhythm (Lang et al., 2002). The arrangement matters clinically as well as physiologically: a conduit whose motor rhythm is coupled to its sensory innervation can register obstruction as pain through the same apparatus that drives its contractions, which is why the analgesic and motor effects of pathway interference are difficult to disentangle at the bedside. Reviews of human ureteral physiology integrate these components and note that the human conduit retains all of them, together with its prostaglandin responsiveness (Canda et al., 2007).

The inhibitory phenotype of PGE2 in the ureter has been documented for six decades and has recently acquired a molecular address. The field opened in 1966 with the observation that prostaglandins inhibit ureteral peristalsis (Boyarsky et al., 1966). A contemporary synthesis of prostaglandin actions across bladder, urethra, and ureter assembled the emerging organ contrast: E-type prostaglandins contract the detrusor while the ureter responds with relaxation at resting tone (Andersson and Forman, 1978b). Direct pharmacological work on the guinea pig ureter then characterized the response and began the receptor dissection (Vermue and Den Hertog, 1987). The relationship between synthesis blockade and motor output, however, is not the simple inverse that a “restore peristalsis” narrative would predict: in isolated sheep ureter rings, the NSAIDs indomethacin and diclofenac themselves inhibit rhythmic motility, reducing frequency and amplitude (Thulesius et al., 1987). The motor effects of synthesis inhibition therefore cut both ways, a point that returns with force in the analgesia debate below. The molecular address arrived only recently, when PGE2 binding to EP2 was shown to relax the rabbit ureter through the cAMP–PKA pathway (Su et al., 2024). The scope of that study deserves explicit statement, because the ureteral row of this review leans on it. It is a single-center study in 25 New Zealand rabbits, with five animals per obstruction-time-point group; randomization of group assignment was reported, but blinding of outcome assessment was not. Isolated ureters relaxed to cumulative PGE2 with an EC50 near 2 × 10⁻^7^ M; the EP2 antagonist PF-04418948 blocked the relaxation while the EP4 antagonist L-161,982 did not; EP2 agonism mimicked a response that EP4 agonism failed to produce; obstructed ureters upregulated mPGES-1 and EP2; and cultured rabbit and human ureteral smooth muscle cells raised cAMP and phosphorylated PKA upon PGE2 exposure. Notably, no *in-vivo* expulsion endpoint was measured: the expulsion promised in the study’s title is a proposal resting on the relaxation data, not a demonstrated acceleration of stone passage. The inhibitory strategy of the ureter thus rests on an evidentiary ladder whose modern rung is single and animal-based: whole-organ phenomenology in the classical era, cross-organ contrast in comparative preparations, and one receptor-defined mechanism in the modern one.

Obstruction rewrites the pathway in both directions at once, producing symptoms through one arm and tissue protection through the other. The inducing signal is mechanical. Stretch engages PI3K signaling to upregulate COX-2 (Owusu-Ofori et al., 2013), and COX-2 rises in both rat and human obstructed ureter (Nørregaard et al., 2006; Nakada et al., 2002). Two temporal patterns follow. In the acute phase after release of bilateral obstruction, COX-2-derived prostanoids accumulate transiently in the renal medulla (Nørregaard et al., 2010). Under endotoxin challenge, the obstructed kidney sustains chronic prostaglandin and thromboxane synthesis (Okegawa et al., 1983). One arm of this response is symptomatic: prostaglandin-saturated ureteral muscle loses propulsive force, the dilated conduit accumulates urine, and stretch-dependent nociceptor discharge follows. The other arm is protective. The genetic evidence is consistent. Disrupting COX-2 exacerbates apoptosis and renal damage during obstruction (Nilsson et al., 2015). mPGES-1 deficiency worsens fibrosis and inflammation in unilateral ureteral obstruction (Luo et al., 2017). The post-obstruction rise of COX-2 attenuates fibrosis in association with EP2 receptor upregulation (Tofteng et al., 2022). EP1 antagonism mitigates fibrosis at early and late stages (Kresse et al., 2022), and fibroblast-intrinsic COX-2 expression drives progressive renal fibrosis (Tsai et al., 2026). Pharmacology adds an apparent contradiction: COX-2 inhibition ameliorates ureteric damage in obstructed uropathy (Chuang et al., 2007) even though genetic COX-2 loss worsens it, a divergence likely rooted in timing, dose, and the cell type in which the enzyme is blocked. The divergence itself suggests a therapeutic window reading: brief pharmacological blockade during maximal inflammatory drive may spare the protective arm that chronic genetic absence removes, a testable interpretation that no published trial has yet exploited. This double bookkeeping mirrors, in reverse, the bladder, where obstruction-induced COX-2 acts predominantly as a pathological amplifier (Section 3); the mirror image prefigures the comparative framework of Section 6.

The clinical armamentarium confirms the pathway’s importance without settling its mechanism. The most recent Cochrane review concludes that NSAIDs are effective for acute renal colic, sustaining their first-line position against placebo, opioids, and spasmolytics (Afshar et al., 2025). Systematic appraisal in the emergency medicine literature reaches the same verdict on both efficacy and safety (Pettit and Welch, 2018), and authoritative syntheses frame prostaglandin blockade as the mechanistic centerpiece of medical expulsive and analgesic therapy (Knoedler and Lieske, 2016). Yet the trial endpoints in this literature are pain scores and analgesic rescue, not ureteral motility, wall edema, or afferent discharge. Clinical success therefore establishes that the pathway matters without establishing which of its branches carries the analgesic effect.

The analgesic role sits inside a wider medical expulsive effort whose pharmacology the pathway also informs. Current medical expulsive therapy for distal ureteral stones rests mainly on alpha-blockade and, in some guidelines, calcium-channel blockade; NSAIDs provide analgesia, not expulsion (Skolarikos, 2018). Reviews of contemporary stone management place prostaglandin blockade at the center of pain control. They also acknowledge that no drug class was designed from ureteral receptor biology (Skolarikos, 2018). The gap is instructive. If EP2 agonism can relax the ureter in experimental models (Su et al., 2024), then receptor-level expulsive therapy becomes a designable successor to empirically assembled regimens: the design target would be the conduit’s own inhibitory strategy rather than systemic sympathetic or calcium-channel targets borrowed from other fields.

Three mechanisms, peristaltic, hydraulic, and afferent, could each contribute to NSAID analgesia (**Figure 3**); they are pharmacologically compatible and may co-occur, and the open question is their relative weight. The peristaltic account starts from the inhibitory phenotype itself: PGE2 suppresses peristalsis (Boyarsky et al., 1966), so synthesis blockade should restore propulsive contractions and help clear the offending stone (Su et al., 2024). Its weaknesses are twofold. Physically, contraction against an unrelieved obstruction raises intraluminal pressure, which should worsen rather than relieve pain; and empirically, NSAIDs can inhibit, not stimulate, isolated ureteral motility (Thulesius et al., 1987), so the motor effect of synthesis blockade *in vivo* is uncertain in direction as well as magnitude. The hydraulic account proposes that NSAIDs reduce PGE2-dependent mucosal edema at the site of impaction, lowering local pressure and improving flow; it is attractive because it explains why the ureteric orifice, where edema concentrates, is the decisive site, but direct measurements of drug-reversed edema in patients are lacking. The afferent account holds that PGE2 sensitizes renal pelvic and ureteral nociceptors and that NSAIDs act as peripheral antihyperalgesics (Lang et al., 2002); it draws strength from the general biology of prostaglandin-dependent nociceptor sensitization but from few direct recordings in the human upper urinary tract. Each account has supporting animal evidence; what none has faced is a human experiment capable of assigning their relative contributions. The evidentiary asymmetries are worth naming. The peristaltic account holds the strongest *in vitro* foundation but the weakest clinical logic, since restored contraction against a stone is mechanically self-defeating. The hydraulic account has the most intuitive clinical fit (urologists observe edema at the impaction site at every procedure) but no quantitative measurement linking NSAID exposure to edema reduction in patients. The afferent account carries the strongest general biology, prostaglandin-dependent nociceptor sensitization is among the best-established findings in pain research, yet the few direct recordings come from animal pelvis and ureter, not human. A concrete design is available, and none of its components requires new technology. Emergency-department patients with computed-tomography-confirmed ureteral stones and active colic would receive standard NSAID analgesia, with pain-intensity trajectory and time to rescue analgesia as clinical endpoints; mechanistic co-endpoints would be acquired at baseline and again at 24–48 hours—ureteral wall thickness and peri-ureteral edema at the impaction site on non-contrast CT, and hydronephrosis grade. Coupling the change in wall thickness to the pain response would test the hydraulic account directly: edema that fails to fall while pain persists would falsify it. Intraluminal pressure is the decisive but least accessible variable. Renal pelvic pressure can be measured in patients, as the Whitaker perfusion test established half a century ago (Whitaker, 1973) and as pressure-sensing guidewires now do continuously during ureteroscopy (Finegan et al., 2026; Tzelves et al., 2026); in unanesthetized colic patients, however, it would have to ride along with clinically indicated stent placement or endoscopy, as a mechanistic sub-study rather than a routine endpoint. Randomizing between NSAID and an active comparator is optional for the clinical question and unnecessary for the mechanistic one, because exposure and response within standard care suffice to assign the weights. No such trial has been run.

**Figure 3:**
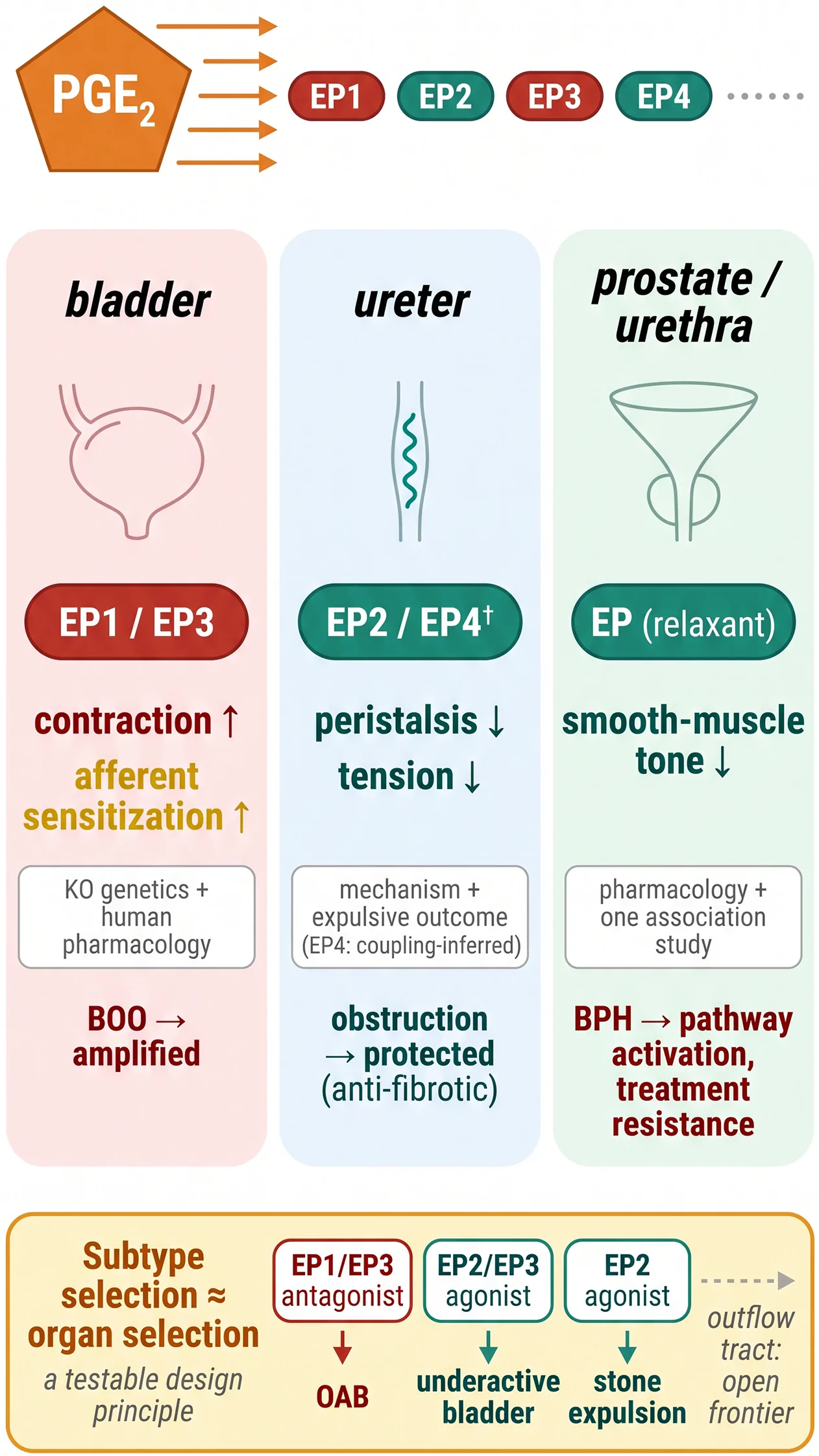
The ureter inhibits where the bladder excites: EP2-dominated deployment and three candidate mechanisms of NSAID analgesia in renal colic. **(A)** The ureteral wall deploys the relaxant limb: EP2 reception relaxes the ureter through cAMP–PKA (demonstrated in the rabbit), while the EP4 contribution remains hypothetical (†); the one direct test found EP4 blockade ineffective (†). PGE2 from the inflamed mucosa around an impacted stone suppresses peristalsis; the conduit dilates proximally. **(B)** Three non-exclusive accounts of NSAID analgesia. Peristaltic: synthesis blockade restores propulsive contractions, yet contraction against unrelieved obstruction raises intraluminal pressure, and NSAIDs inhibit rather than stimulate isolated ureteral motility. Hydraulic: reduced PGE2-dependent mucosal edema would lower local pressure, but drug-reversed edema has not been measured in patients. Afferent: withdrawal of prostaglandin-dependent nociceptor sensitization rests on strong general biology but few human upper-tract recordings. A trial pairing intraluminal pressure or wall thickness with pain scores would assign their relative contributions.

The inhibitory strategy thus ends where it began, at the bedside. Translation has already succeeded in one direction: NSAIDs, acting on this pathway, are established first-line therapy for renal colic (Afshar et al., 2025). A second direction is opening, with EP2 agonism proposed to relax the ureter and, by that route, promote stone passage (Su et al., 2024). Three gaps constrain what either direction can deliver. At human verification, EP receptor distribution in the human ureter remains unmapped at subtype resolution, with human physiology syntheses still leaning on rodent pharmacology (Canda et al., 2007). At mechanism resolution, no trial has paired NSAID analgesia with concurrent measurement of motility, edema, and afferent markers, so the three-mechanism contest of this section persists by default. At precision, patient factors such as stone size and impaction site should, in principle, select for peristalsis-promoting versus edema-reducing regimens, but no stratified analysis has tested the idea. Resolving these gaps would turn a drug class that succeeded by trial into a mechanism-guided one.

## 5 The prostate and urethra: EP-mediated relaxation and outflow regulation

The outflow tract plausibly completes the triad with a relaxation strategy. In prostate and urethra, EP-dominated reception lowers smooth muscle tone and therefore outflow resistance, an arrangement that cooperates functionally with bladder emptying instead of competing with it. Human tissue experiments established the relaxant response early, rodent and rabbit pharmacology assigned it to inhibitory prostanoid receptors, and clinical observations in benign prostatic hyperplasia (BPH) report the pathway measurably activated, most strongly in patients who fail standard medical therapy—an association whose causal direction is untested. The evidence base here is the thinnest of the three organs; this section reports that fact rather than concealing it, because the gap itself defines the research agenda.

Prostatic smooth muscle carries an inhibitory prostanoid receptor population that has been characterized pharmacologically across four decades. Early comparative work found that prostaglandin E activity dominates the prostanoid responses of urinary tract smooth muscle *in vitro* (Klarskov et al., 1983a), and systematic testing of individual prostaglandins on dog bladder and urethral preparations confirmed organ-specific directionality, with relaxation prominent in the outflow segments (Mutoh et al., 1983). A broad agonist survey of rabbit lower urinary tract smooth muscle placed prostaglandins among the active regulators of prostatic tissue tone (Sudoh et al., 1997). The receptor-level dissection arrived later: in the rat prostate, the prostanoid receptor mediating inhibition of smooth muscle contractility has been pharmacologically characterized, establishing the relaxant identity of the response and its compatibility with a cAMP-coupled subtype (Tokanovic et al., 2010). On the synthesis side, both COX-1 and COX-2 are expressed in the human prostate, so the human tissue contains the machinery to generate the mediator it responds to (Kirschenbaum et al., 2000).

The urethra, shared by both sexes, relaxes to PGE2 in human tissue and retains the response along its length. The founding experiment predates receptor nomenclature: in isolated human preparations, PGE2 contracted bladder body strips while relaxing urethral tissue from the same patients (Andersson et al., 1977), and local application of PGE2 in women lowered intraurethral pressure *in vivo* (Andersson et al., 1978a). Systematic *in vitro* pharmacology of human male urethral smooth muscle provides the modern benchmark against which any EP-subtype claim must be tested (Kedia et al., 2013). Contemporary work is beginning to examine urethral smooth muscle at ion-channel resolution, as with anoctamin-1 channels (Drumm and Gupta, 2026), but the intersection of prostanoid signaling with that electrical machinery remains unexplored, and no subtype-resolved EP map of the human urethra exists.

The relaxant deployment also operates within a crowded neuropharmacology. Non-adrenergic, non-cholinergic nerves mediate relaxation of trigone, bladder neck, and urethra (Klarskov et al., 1983b), vasoactive intestinal polypeptide relaxes detrusor, trigone, bladder neck, and urethral smooth muscle from human and pig *in vitro* (Klarskov et al., 1984), and ATP-with-VIP relaxant responses have been mapped segment by segment in hamster proximal urethra (Pinna et al., 1998). Where prostaglandins fit into this peptidergic control is simply unknown: the interactions between EP-mediated relaxation and NANC relaxant innervation have never been systematically tested in any species, which leaves the third strategy neurologically uncharacterized even at the descriptive level.

In benign prostatic hyperplasia, the pathway is not silent: a single tissue-comparison study reports it detectably activated, most strongly in patients who fail standard medical therapy. The constitutive expression of COX isoforms in human prostate (Kirschenbaum et al., 2000) sets the baseline. The decisive observation is comparative: prostate tissue from patients who failed standard medical therapy for BPH shows the prostaglandin pathway significantly activated relative to treatment-responsive tissue, linking pathway tone with therapeutic resistance (Jin et al., 2021). Animal models point in the same direction: probenecid ameliorates testosterone-induced BPH in rats with implications for PGE-2 signaling (Abdel-Fattah et al., 2023). Two cautions bound the interpretation. The clinical finding is an association, not a demonstrated cause; pathway activation may accompany hyperplasia or inflammation rather than drive resistance. And no interventional trial has yet targeted the pathway in BPH, so clinical weight here means plausibility plus one strong correlation, not proven mechanism.

The relaxation strategy thus rests on human tissue evidence, rodent receptor pharmacology, and one strong clinical association. No EP-directed therapy for BPH or urethral dysfunction has reached the clinic, leaving the outflow tract as the strategy with the clearest gap between biological evidence and therapeutic exploitation. Three deficiencies dominate the agenda. At the atlas level, subtype-resolved EP expression in human prostate and urethra has never been systematically mapped, in contrast to the detrusor, where human pharmacology is at least partially established. At the mechanistic level, no outflow-tract-specific genetic model exists, so the third strategy alone lacks knockout verification that the bladder and ureter strategies already possess. At the sex-difference level, female urethral prostanoid pharmacology is close to empty, resting on pressure studies from the 1970s (Andersson et al., 1978a). The emptiness is structural. Surgical access to normal female urethral tissue is rare, animal studies default to males, and the male prostatic preparation dominates the outflow literature by historical momentum. Filling these gaps in the order given (mapping first, mechanisms second, sex differences throughout) would bring the third strategy to the evidentiary standard of the first two.

## 6 One axis, three strategies: a comparative synthesis

Across the three organs, one signaling system resolves into a small set of deployment rules consistent enough to be treated as a design principle, provided the principle is stated together with its exceptions and its evidentiary limits. The bladder excites through EP1 and EP3; the ureter quiets through EP2 and EP4; the outflow tract relaxes through EP-dominated reception. **Table 2** assembles the matrix, and **Figure 4** condenses it into a single view. This section reads the matrix four ways: as a regularity, as the product of plausible functional selection, as a target that pathology reprograms but does not erase, and as an evidentiary record whose inconsistencies owe as much to method as to biology.

**Table 2:** One axis, three strategies: dominant EP deployment, effect direction, and evidence grade across urinary tract organs.

Organ	Dominant subtype(s)	Effect direction	Cellular substrate	Evidence type	Grade
Bladder	EP1/EP3	Contraction + afferent sensitization	Detrusor/ICC/urothelium/afferents	KO genetics + cross-species pharmacology incl. human	★★★
Bladder	EP2/EP4	Relaxation (low-dose limb)	Detrusor/mucosa	Pharmacology (biphasic component)	★★
Bladder (pathology)	COX-2 ↑/EP4 re-expression	Amplified excitation	Whole wall	BOO/inflammation models + human tissue	★★
Ureter	EP2	Relaxation, peristaltic inhibition	Smooth muscle	Molecular mechanism (cAMP–PKA, rabbit model)	★★
Ureter	EP4	Relaxation (hypothesis)	—	Coupling inference; direct rabbit testing found EP4 blockade ineffective	★†
Ureter (pathology)	COX-2/mPGES-1/EP2 ↑	Protective (anti-fibrotic)	Ureteric + renal tissue	KO/pharmacologic divergence	★★
Prostate	EP (relaxant, cAMP-compatible)	Relaxation	Prostatic smooth muscle	Rodent receptor characterization	★
Urethra	EP (relaxant)	Relaxation	Urethral smooth muscle	Human tissue *in vitro* (1977–)	★★
Outflow (pathology)	Pathway activation	Association with treatment resistance	Human BPH tissue	Single clinical study	★

Evidence grades: ★★★, knockout genetics plus human data; ★★, strong mechanistic or human evidence along a single line; ★, single pharmacological or association study; †, hypothesis status (the ureter EP4 row: inferred from coupling logic, with direct rabbit testing finding EP4 blockade ineffective). Each row is anchored to the studies cited in the corresponding text section; no new references are introduced here.

**Figure 4:**
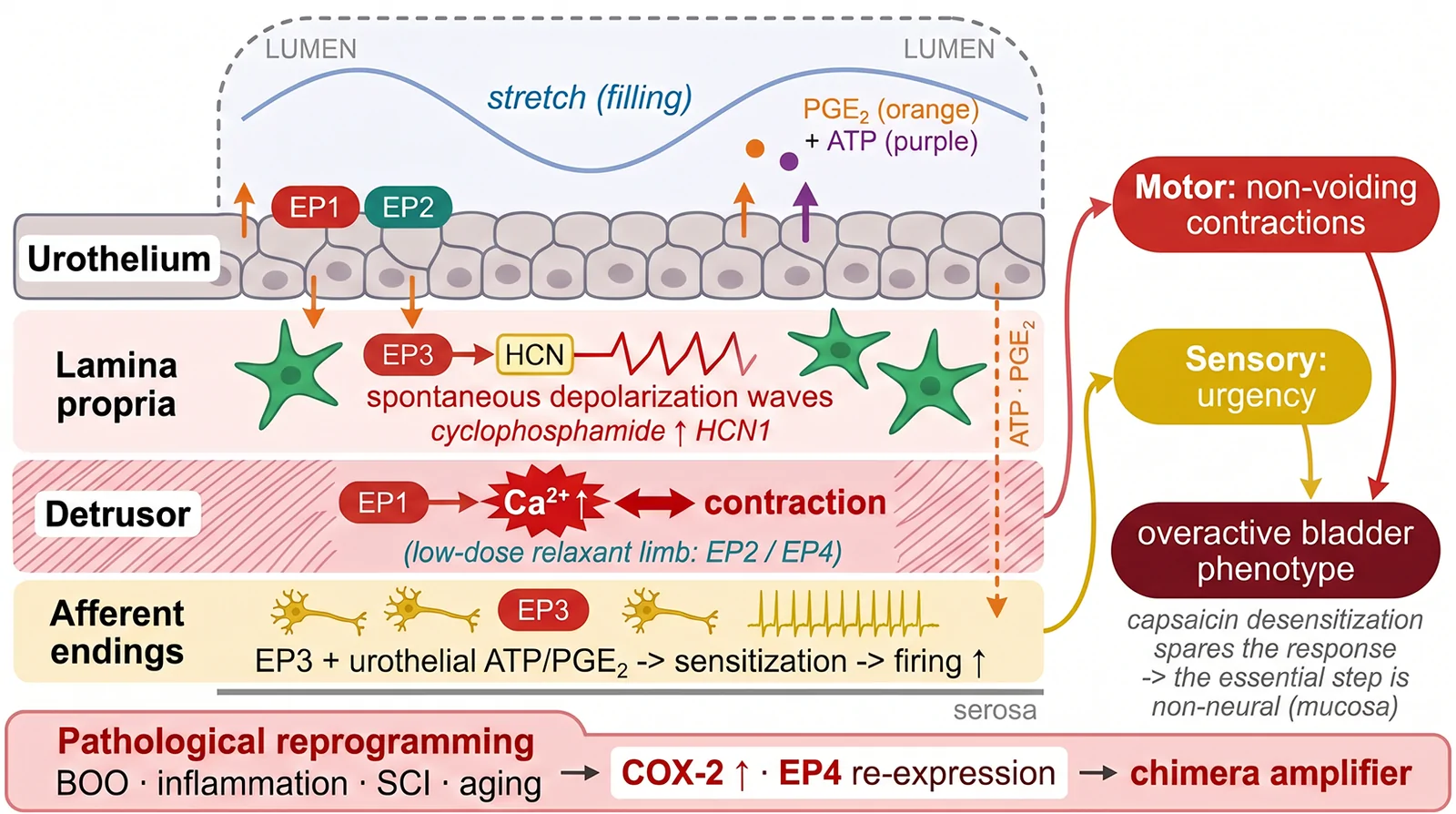
One signaling system, three organ-specific strategies: receptor-subtype deployment across the urinary tract. Top: one PGE2 signal feeds four EP subtypes with opposing coupling directions (red, excitatory; teal, inhibitory). Columns: the bladder excites through EP1/EP3 (contraction with afferent sensitization; knockout genetics plus human pharmacology); the ureter quiets through EP2 (cAMP–PKA relaxation in a rabbit model; expulsion itself untested), with EP4 shown as hypothesis (†); the outflow tract relaxes through EP-dominated reception (pharmacology plus one association study). Pathology lines: obstruction amplifies the bladder’s excitatory deployment, turns protective and anti-fibrotic in the ureter, and tracks treatment resistance in benign prostatic hyperplasia. Bottom: subtype selection approximates organ selection—EP1/EP3 antagonism for overactive bladder, EP2/EP3 agonism for underactive bladder, EP2 agonism for stone expulsion—with the outflow tract an open frontier.

The matrix itself is compact: each organ’s dominant response follows the coupling direction of its dominant receptor subtype. In the bladder row, contraction and afferent sensitization rest on EP1 and EP3, a conclusion that survives both genetics, since EP1-deficient and EP3-deficient mice display distinct loss-of-function phenotypes (Schröder et al., 2004; McCafferty et al., 2008), and cross-species pharmacology, since the same contractile dominance holds in rat, macaque, and human detrusor (Root et al., 2015). In the ureter row, relaxation and peristaltic quieting rest on EP2, which now carries a defined cAMP–PKA mechanism in the rabbit (Su et al., 2024), while the EP4 contribution remains a coupling-based hypothesis. In the outflow row, relaxation dominates, with receptor-level characterization so far confined to rodent prostate (Tokanovic et al., 2010). The organ contrast was visible to the first investigators who compared segments in one preparation (Andersson and Forman, 1978b) and has hardened rather than dissolved as human data accumulated (Root et al., 2015). The three rows do not rest on equal evidentiary footing, and the matrix states which is which: the bladder row carries knockout genetics plus human pharmacology, the ureter row carries molecular mechanism plus clinical outcome, and the outflow row carries pharmacology without genetics and a single strong clinical correlation. On current evidence, then, the triad is best read as a dyad with a plausible third member: two strategies with established mechanisms, and one whose case is strong but unsealed. Two qualifications keep the matrix honest. It is a statement of dominant weights, not of exclusive expression: the bladder retains relaxant components that surface at low concentrations, and isolated ureters can report contraction under some conditions. The matrix is also deliberately PGE2-centric. The urinary tract produces other prostanoids with their own receptors: prostaglandin D2 acts through DP receptors in guinea pig bladder with inhibitory consequences (Guan et al., 2014), and human detrusor contracts robustly to thromboxane mimetics (Palea et al., 1998). A complete receptor economy of the urinary tract would need rows the present synthesis does not attempt. And the matrix rests on functional inference, since no study has measured all four receptor subtypes in all three organs by a common assay; within the ureter row, the EP4 assignment rests on coupling inference; the one direct test found EP4 blockade ineffective against PGE2-induced relaxation in the rabbit ureter (Su et al., 2024).

Why one mediator and three deployments? The most economical explanation is functional selection: each organ’s mission favors the coupling direction that serves it. The bladder must convert a filling signal into forceful, periodic emptying, and calcium-mobilizing reception (Coleman et al., 1994) aligns excitation with that mission. The ureter must move urine by peristalsis at low pressure, and cAMP-coupled reception (Breyer et al., 2001) maintains the low resting tone the mission requires. The outflow tract must open in coordination with voiding, and a relaxant deployment lowers resistance at exactly that moment. The appearance of COX-2 during bladder development (Park et al., 1997) suggests that the machinery is installed early, before adult functional demands, consistent with deployment rather than afterthought. The hypothesis accommodates its own exceptions: biphasic concentration responses mean that deployment is a weighting of receptor populations, not an exclusion of subtypes, and the coexistence of multiple receptor populations within one human tissue (Palea et al., 1998) allows local cellular needs to override the organ-level rule. It must be stated plainly that this is a hypothesis, not a demonstrated mechanism; it earns its place by being testable, and the closing paragraphs of this review specify the tests. A decisive test would be developmental time-series interference: if forced receptor reassignment in one organ’s smooth muscle fails to shift its PGE2 response toward the donor organ’s pattern, the selection account fails.

Disease stress-tests the principle, and the stress test passes in an instructive way: pathology reprograms the axis without erasing organ-specific direction. In the obstructed or inflamed bladder, COX-2 induction and EP4 re-expression (Beppu et al., 2011) amplify the excitatory output the organ already favors. In the obstructed ureter, the same enzyme family is upregulated (Tofteng et al., 2022) yet its role inverts to protection, countering fibrosis and preserving microvasculature. Genetic COX-2 loss worsens obstruction damage (Nilsson et al., 2015) even as pharmacological COX-2 inhibition improves ureteric outcomes (Chuang et al., 2007). The divergence is attributable to timing and cell type, not to organ-level logic. In the hyperplastic prostate, pathway activation tracks therapeutic resistance in the single study that has measured it (Jin et al., 2021). Read together, the three disease states show the same system being reweighted in three organ-specific directions. Each reweighting is also informative about its organ: in the bladder it identifies the axis with symptom amplification, in the ureter with tissue defense, and in the prostate with treatment resistance, so the pathology shows both that deployment is preserved and what each deployment is for. Pathology does not overwrite the design principle; it stress-tests it line by line, and each line holds.

How much of the apparent inconsistency across studies is real biology, and how much is method? Five variables do most of the damage. First, preparation: isolated strips strip away neural and mucosal input, and retaining or removing the mucosa changes the apparent contractile response of the same tissue (Stromberga et al., 2020). Second, species: receptor weights differ among rabbit, guinea pig, rat, and human, which is precisely why cross-species comparisons that include human tissue carry the evidentiary load (Root et al., 2015; Palea et al., 1998). Third, ligand selectivity: a compound selective in one species background can lose selectivity in another, so receptor assignments inherited from rodent pharmacology require human re-verification (Palea et al., 1998). Fourth, concentration range: biphasic curves make the reported direction of an effect a function of the range tested. This ambiguity has shadowed the detrusor literature since the classical era, when low-dose relaxation and high-dose contraction coexisted in the same preparations (Gotoh et al., 1986) and single-range studies inherited whichever limb they sampled. Fifth, and most telling, the *in vitro*–*in vivo* divide: the same drug class that inhibits isolated ureter rings in the bath (Section 4) delivers analgesia in patients (Afshar et al., 2025); the two findings sit adjacent rather than contradictory once the preparation variable is named. Most contradictions in the matrix dissolve into one or two of these variables; the residue that survives methodological control is the reprogramming itself. A practical remedy costs little: papers reporting PGE2 effects on urinary tract preparations could state, as a matter of routine, the segment and species, whether mucosa was retained, the concentration range relative to the biphasic curve, and whether the preparation was isolated or *in vivo*. A reporting standard of that kind would let the existing four-decade literature be re-mined for the true organ contrasts, converting an accumulated inconsistency into a distributed dataset.

Sex runs through all three organs and is handled unevenly by the underlying literature. The prevalence landscape is sex-skewed from the outset: among adults with overactive bladder, women carry the larger share (Zhang et al., 2025), symptomatic stone disease is more common in men (Scales et al., 2012), and BPH is by definition confined to men (Egan, 2016). The mechanistic base, by contrast, is sex-blind by default rather than by design: the rodent instillation, knockout, and antagonist studies that anchor the bladder and ureter strategies were performed predominantly in male animals, and sex-stratified EP receptor data do not exist for either organ at the resolution this review assembles. The outflow tract inverts the pattern: female urethral prostanoid pharmacology is nearly empty (Section 5), while the male prostatic preparation dominates by historical momentum. Neither imbalance serves the framework. An organ-by-receptor matrix built largely on male tissue cannot be assumed to transfer to female tissue, and a void where female urethral pharmacology should be leaves the third strategy half-mapped. Reporting sex, estrous cycle state in rodents, and hormonal status in human tissue donors would cost little; until such reporting becomes routine, sex remains the least examined of the variables that shape the matrix.

The design principle carries a translational corollary: subtype selection approximates organ selection. An EP1/EP3 antagonist should, other things equal, act as a bladder-directed agent; an EP2 agonist as a ureter-directed one; an outflow-directed strategy should exploit the relaxant deployment. Section 7 develops this corollary pharmacologically. Three gaps bound what the principle can currently claim. At the atlas level, no cross-organ, single-cell-resolved map of EP expression exists, so the matrix’s weights are inferred from function rather than measured as transcript or protein. At the hypothesis level, the functional-selection account awaits two decisive experiments: parallel conditional knockouts driving the same receptor deletion in smooth muscle of all three organs, and developmental time-series showing whether deployment precedes function. At the toolkit level, ligand sets whose selectivity is verified across species remain incomplete. Closing these gaps in order, first mapping, then testing, then equipping, would elevate the three strategies from a descriptive pattern to a mechanistic rule of urinary tract biology.

## 7 Therapeutic targeting of the PGE2–EP axis

Therapeutic exploitation of the axis comes in two grades. Synthesis-level blockade, embodied by NSAIDs, is empirically successful but subtype-blind, and the mPGES-1 experience warns that blocking one synthetic node can redirect mediator flow rather than silence it (Wang et al., 2006). Receptor-level targeting, by contrast, converts the design principle of Section 6 into an organ-selection problem: choosing a subtype chooses an organ. This section takes stock of what each grade has delivered, what the receptor-grade inventory actually contains, and where the four major indications stand.

Several established drugs already act on the pathway, but as incidental inhibitors rather than designed ones. NSAIDs hold first-line position in renal colic on the strength of clinical evidence (Afshar et al., 2025), yet they act at the synthesis level, without subtype resolution, and their analgesic mechanism remains an open contest (Section 4). Intravesical botulinum toxin suppresses COX-2 and EP4 expression together with non-voiding contractions (Chuang et al., 2009), so part of its benefit plausibly rides on pathway down-regulation. Antimuscarinics reduce urothelial ATP and PGE2 release (Yokoyama et al., 2011), giving OAB pharmacology a sensory arm it was never designed around. The alpha1-blocker naftopidil lowers intravesical prostaglandin levels while reducing non-voiding contractions in obstructed rats (Akino et al., 2019), and prostaglandin-targeting agents shift autonomic indices in experimental partial obstruction as well (Dobrek et al., 2016), overlaps between standard therapy and the axis that pass unexploited. The common pattern is that pathway inhibition is an incidental component of drugs aimed at other targets; target validation, in effect, has been done passively. The increment available to designed targeting is therefore not proof of principle, which the incumbent drugs already supply, but selectivity.

The receptor-grade inventory is real but preclinical, and its strongest asset is the ONO-8055 record in underactive bladder; **Table 3** grades each entry by evidence level. On the antagonists side, a novel EP1 receptor antagonist showed urodynamic effects in normal rats and in rats with bladder outlet obstruction (Lee et al., 2007), and selective EP4 antagonists reduced detrusor overactivity in cyclophosphamide and PGE2 models (Chuang et al., 2010, Chuang et al., 2012). On the agonist side, ONO-8055, the dual EP2/EP3 agonist, improved voiding in a rat neurogenic model (Sekido et al., 2016) and a primate surgical model (Matsuya et al., 2018), with extended characterization to follow (Sekido et al., 2020a). This makes the “augment contractility” direction the most completely documented receptor-level strategy in the urinary tract. That direction also has a forgotten human precedent: combined PGE2 and bethanechol was administered for detrusor underactivity two decades ago (Hindley et al., 2004), an untargeted precursor of the same idea that ONO-8055 now executes with subtype resolution. EP2 agonism to relax the ureter extends the same logic to the conduit (Su et al., 2024). Safety enters the ledger as well: the EP4 antagonist ASP7657 was advanced for its renal safety profile in systemic contexts (Mizukami et al., 2019), a reminder that subtype targeting must clear organ-external liabilities. The inventory’s shared limitation is stark: every entry rests on animal or ex vivo evidence, and none has been verified in human tissue at the standard the detrusor literature already sets (Root et al., 2015).The most advanced candidate, ONO-8055, entered clinical evaluation in Europe, according to the sponsor’s development pipeline disclosures (Ono Pharmaceutical Co., Ltd., unpublished company information, accessed 27 September 2026; we could identify no peer-reviewed report or public trial registration documenting the program), before its development was discontinued. Even as a corporate record that cannot be verified beyond the company’s own disclosures, it shows that clinical entry is possible and that the underactive bladder indication remains difficult.

**Table 3:** Two targeting grades mapped onto four indications: evidence and development status.

Indication	Targeting grade	Subtype strategy	Representative candidate(s)	Evidence level	Development status
Renal colic (analgesia)	Synthesis-level	COX inhibition (non-selective)	NSAIDs (ketorolac, diclofenac, indomethacin)	Cochrane-confirmed efficacy	First-line standard care
Stone expulsion	Receptor-level	EP2 agonism	(preclinical candidate; rabbit relaxation model, expulsion untested)	Animal model only	Preclinical
Underactive bladder	Receptor-level	EP2/EP3 dual agonism	ONO-8055	Rat + primate models	Reached phase I (Europe); development discontinued
Underactive bladder (historical)	Non-selective	PGE2 instillation + bethanechol	—	Small uncontrolled human series	Historical, superseded
Overactive bladder	Receptor-level	EP1 (or EP4) antagonism	PF-2907617-02; MF191	Rat urodynamics	Preclinical
OAB/cystitis (adjacent)	Synthesis-level	COX-2 inhibition	Botulinum toxin (indirect COX-2/EP4 ↓); celecoxib-class (prevention, experimental)	Human (BoNTX clinical)/animal (recurrence prevention)	BoNTX standard for refractory OAB; COX-2 prevention experimental
BPH/outflow	—	None defined	—	Single association study	Open frontier

The ONO-8055 phase I status reflects unpublished company pipeline disclosure (Ono Pharmaceutical Co., Ltd.; accessed 27 September 2026) rather than peer-reviewed publication or public trial registration, and should be verified against the sponsor’s current records at the time of reading. NSAID agent names are those appearing in the cited Cochrane and systematic reviews. BoNTX, botulinum toxin; OAB, overactive bladder; BPH, benign prostatic hyperplasia.

Mapped onto four indications, the subtype–organ correspondence yields one established success, two credible prospects, and one open frontier. In OAB, where current drugs offer only moderate benefit, bladder-directed EP1/EP3 antagonism has animal urodynamic support but no clinical program. In underactive bladder, the indication least served by pharmacotherapy (Moro et al., 2022; Juszczak and Drewa, 2016), the ONO-8055 record (Sekido et al., 2016; Matsuya et al., 2018; Sekido et al., 2020a, b) makes EP2/EP3 agonism the best-documented prospect, now carrying the cautionary weight of a discontinued clinical program documented only by company disclosure (see above). In renal colic and stone expulsion, synthesis-level blockade is already standard care, and receptor-level EP2 agonism is the mechanistically guided successor candidate (Su et al., 2024). In BPH and the outflow tract, no receptor-level candidate exists at all; the observation that COX-2 inhibition restores urothelial integrity after obstruction (Erdogru et al., 2005) and that pathway activation tracks treatment resistance (Section 5) defines the frontier without yet arming it.

Three steps would move this inventory toward the clinic. The first is cross-species ligand verification: every subtype assignment inherited from rodent pharmacology requires re-establishment in human tissue, and the human detrusor receptor map (Root et al., 2015) shows what that standard looks like. The second is trial design with mechanism endpoints: the renal colic literature demonstrates how clinical success without mechanistic endpoints leaves a three-way contest unresolved (Section 4, where a concrete adjudicating design is specified), and future programs should pair subtype-selective agents with motility, edema, or afferent biomarkers from the outset. Urinary prostaglandin metabolites offer one ready candidate, since urinary PGE-M reports systemic pathway output and falls when the synthetic arm is deleted (Wang et al., 2006); adapting such markers to organ-localized readout is an unsolved but tractable problem. The third is combination logic: because the design principle assigns different subtypes to different organs, rational combinations, an EP1 antagonist with an EP2 agonist for example, become addressable rather than *ad hoc*. Combinations may also cross mechanism classes: prostaglandin-directed agents and phosphodiesterase inhibitors have been discussed as complementary handles on the same storage pathology (Rahnama’i et al., 2013), and in recurrent cystitis, COX-2 inhibition prevented chronicity in experimental models (Hannan et al., 2014), raising the prospect of pathway prophylaxis rather than pathway rescue. Whether the principle converts into medicine is, in this sense, a matter of execution rather than of doubt; Section 8 returns to what execution requires.

## 8 Discussion

This review set out to answer a simple question with a scattered literature: why does one mediator excite the bladder, quiet the ureter, and relax the outflow tract? The answer assembled here is a qualified yes: the PGE2–EP axis is best described as a single signaling system deployed in three organ-specific strategies—excitation through EP1 and EP3 in the bladder, inhibition through EP2 in the ureter, and relaxation through EP-dominated reception in the outflow tract—and pathology appears to reweight this deployment rather than erase it. The qualification is not decorative. The three strategies rest on unequal evidence—knockout genetics and human pharmacology for the bladder, a single mechanistic animal study for the ureteral receptor assignment, rodent pharmacology and one clinical association for the outflow tract—so the triad currently stands as two established strategies plus a plausible third, and the receptor weights everywhere are inferred from function rather than measured as abundance. Stated with those limits attached, the pattern does three kinds of work at once. It explains, mechanistically, why six decades of segment-by-segment studies kept reporting opposite effects for the same mediator. It supplies a research template: the organ-by-receptor matrix converts scattered observations into a set of weights that can be measured, compared, and perturbed. And it turns therapy into an organ-selection problem—choosing a subtype chooses an organ—which is the logic on which the therapeutic mapping of Section 7 rests.

Realizing that logic faces challenges that layer outward from the bench. The innermost layer is the model: receptor weights and ligand selectivity are species-dependent, so assignments inherited from rodent pharmacology fracture at the human boundary, and the *in vitro*–*in vivo* divide can invert a drug’s apparent motor effect, as the ureteral NSAID case showed. The next layer is data integration: no cross-organ, single-cell-resolved map of EP1–EP4 expression exists, so the matrix’s weights remain functional inference; the field measures what prostaglandins do far more precisely than where the receptors are. The third layer is methodological: parallel conditional knockouts, deleting the same receptor in the smooth muscle of all three organs, have never been built, although they are the decisive test of the functional-selection hypothesis, and human tissue verification remains at the standard the detrusor literature set decades ago. The outermost layer is computational. Machine learning over single-cell and spatial transcriptomes could assign EP expression to cell types across organs automatically. Cross-species models could predict whether a ligand’s selectivity survives transfer before a synthesis program begins. Mechanism-endpoint modeling could rescue trial interpretation from the endpoint poverty that left renal colic analgesia without an assigned mechanism. Concretely, an EP atlas built this way would replace the functional inference behind the organ-by-receptor matrix with measured receptor weights, turning the matrix from a summary of past experiments into a sampling frame for the next ones. This outermost layer is currently empty, which makes it the largest opportunity rather than the least important gap.

These layers resolve in order. Maps will replace inference, and the replacement matters most for the outflow row, whose weights are today the most poorly constrained. Parallel knockouts will confirm or break the functional-selection hypothesis at its strongest point, the shared developmental installation of the machinery. Verified ligands will carry the principle into trials whose biomarker arms return quantitative receptor weights, not anecdotes. Each translational step returns evidence: human subtype data from those trials will flow back to confirm, refine, or rewrite individual rows of the matrix, and the outflow row, currently the thinnest, is the one most likely to be rewritten. The principle’s value lies exactly in being falsifiable in this way. If it survives, urology gains a mechanistic rule for organ-directed pharmacology spanning storage, transport, and emptying. If it does not, the failure will itself localize where urinary tract smooth muscle diverges from simple receptor-deployment logic, and the field will have learned the shape of that divergence. Either outcome advances the science.
