# Supplementary material for: The PGE2–EP receptor axis in urinary tract smooth muscle: one signaling system, three organ-specific strategies
Source: Front Physiol. 2026 Oct 28;17:1977562. doi: 10.3389/fphys.2026.1977562 (PMC13752544; doi:10.3389/fphys.2026.1977562)
Supplement: Supplementary file 1 [file DataSheet1.pdf]

Literature search and selection flow

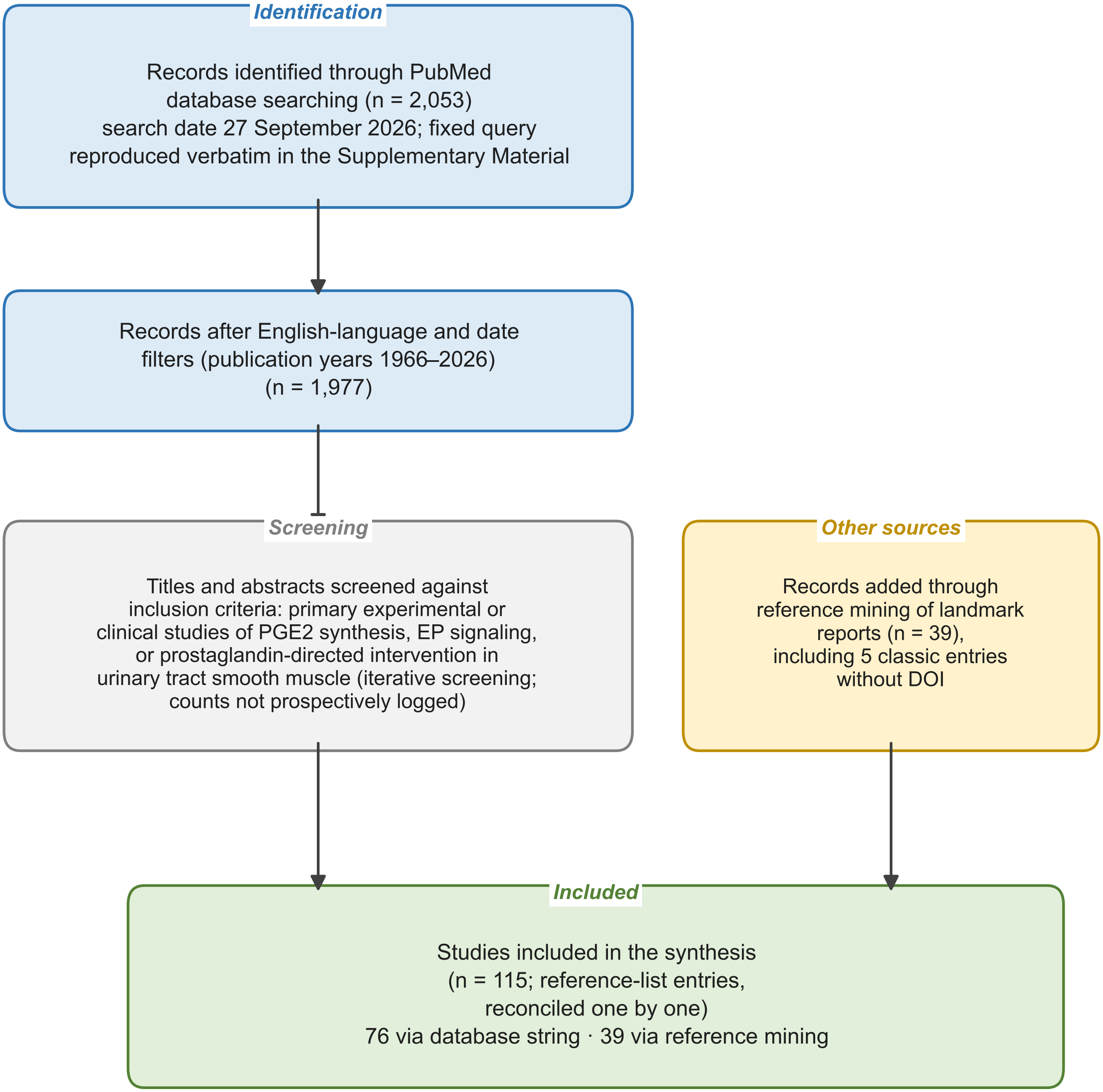

Database: PubMed (NLM E-utilities). Screening was iterative rather than prospectively logged, in keeping with a narrative review; databases, date of last search, query, and inclusion criteria are reported in full in the Methods paragraph of the Introduction and reproduced in the Supplementary Material.
